# Supplementary figures and images for: Effect of a communication robot in the prevention of postoperative delirium in older persons: A randomized controlled trial
Source: PLoS One. 2025 Jul 29;20(7):e0327868. doi: 10.1371/journal.pone.0327868 (PMC12306737; doi:10.1371/journal.pone.0327868)

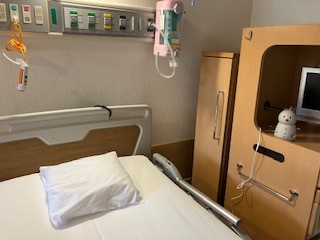

Supplement: S3 Appendix — (JPG) [file pone.0327868.s003.jpg]
