# Supplementary material for: Effect of a communication robot in the prevention of postoperative delirium in older persons: A randomized controlled trial
Source: PLoS One. 2025 Jul 29;20(7):e0327868. doi: 10.1371/journal.pone.0327868 (PMC12306737; doi:10.1371/journal.pone.0327868)
Supplement: S1 File — (DOC) [file pone.0327868.s004.doc]

Independent clinical research

**Intervention study of communication robots on the effectiveness of delirium prevention**

Research implementation plan

Research director: Koubun Imai, Department of Mental Health, Hitachi General Hospital

Date of creation: March 18, 2022 Draft plan version 0 created

**Table of Contents**

0. Overview

1. Background of the study

2. Study objectives

3. Target patients and eligibility criteria

4. Study methods

5. Observation and testing items

6. Expected benefits and disadvantages (side effects)

7. Evaluation items (endpoints)

8. Handling of adverse events

9. Discontinuation criteria for individual subjects

10. Study duration

11. Target number of cases and rationale for setting it, and statistical analysis method

12. Consideration of human rights of subjects and method of protecting personal information

13. Response to and compensation for health damage to subjects

14. Cost burden for subjects

15. Record keeping and publication of study results

16. Research funding and conflicts of interest

17. Study implementation structure

18. References and literature list

1. **Overview**

Delirium is a disorder of attention and consciousness that develops rapidly and fluctuates in severity, and is also accompanied by cognitive impairment. In some cases, patients become restless and refuse treatment, which can lead to various adverse effects such as extended hospital stays, increased hospital costs, poor prognosis, and exhaustion for medical professionals, creating a vicious cycle. However, since there is still no evidence-based treatment, it is an urgent task to find an effective prevention method.

The purpose of this study is to compare a group of high-risk delirium patients who receive regular delirium countermeasures with a group who receive the same countermeasures plus a communication robot intervention, and to verify the effectiveness of communication robots in preventing the onset of delirium. At the same time, if the subject is a dementia patient, changes in behavior and psychological symptoms will be investigated. In addition, a questionnaire survey will be conducted on nurses who actually treated patients in this study to explore the functions required for communication robots.

The subjects will be patients who are determined to be high-risk for delirium in screening at the time of admission, excluding patients who had severe psychiatric symptoms before admission and those who drank a lot of alcohol.

The study will be conducted at Hitachi General Hospital alone. This is an open-label randomized controlled trial in which subjects are randomly assigned to a control group that receives regular delirium prevention measures, and a group that receives the same measures plus an intervention using a communication robot with a chat function (NEKORIKO's BOCCO emo LTE model), and the evaluation items are compared between the groups. The observation period is from consent until discharge, but if the hospitalization period is three weeks or longer, it will end three weeks after hospitalization. In addition, after the subjects are discharged from the hospital, a questionnaire survey will be conducted for the nurses who cared for the patients in this study.

The primary evaluation item is the number of cases of delirium during the hospitalization period up to three weeks after hospitalization, which will be determined using the Japanese version of the ICDSC. The secondary evaluation items are: 1) in the case of dementia, changes in behavioral and psychological symptoms using the Japanese version of the DBD scale, and 2) the burden of using the communication robot and improvements to the robot, based on a questionnaire survey of nurses who actually cared for patients in this study.

The target number of cases to be conducted is 72 cases in each group (144 cases in total). The study period is scheduled to run from October 1, 2022 to March 31, 2025 (registration deadline: September 31, 2024), but registration will be closed once the target number of cases is reached.

**1. Background of the study**

Delirium is a disorder of attention and consciousness that develops suddenly and fluctuates in severity, and is also accompanied by cognitive impairment. In some cases, patients become restless and refuse treatment, which can lead to various adverse effects such as extended hospital stays, increased hospital costs, poor prognosis, and exhaustion for medical professionals, which can lead to a vicious cycle. Delirium is one of the major problems in acute care. As a psychiatrist at a general hospital, the principal investigator has long been working on delirium countermeasures, but what he has realized about delirium is that there is still no effective treatment for it, and how important it is to prevent its onset. With the revision of medical fees in 2020, a new "delirium high-risk patient care surcharge" was established, and evaluation has begun for screening of high-risk patients for delirium, preventive measures centered on non-pharmacological therapy, and early detection of delirium. However, since there is still no evidence-based treatment, the current situation is that multiple preventive measures are combined, and it is an urgent task to find more effective preventive methods. On the other hand, the Ministry of Economy, Trade and Industry and the Ministry of Health, Labor and Welfare have been supporting the development and demonstration of nursing robots for some time. The introduction of nursing robots has been verified in areas such as transfer assistance, mobility assistance, excretion assistance, bathing assistance, and monitoring of people with dementia. Among nursing robots, communication robots are those that have communication as a purpose or means. Old age and dementia are known to be major risk factors for delirium. Stuffed animal-type communication robots, including the seal-type robot PARO, are currently being researched for their effectiveness in dementia care, and there are high expectations for their clinical application. However, the price of PARO is very high, at around 400,000 yen. In addition, stuffed animal-type robots in general are difficult to disinfect because of the emphasis on tactile sensation, and with the COVID-19 problem showing no signs of ending, there is a demand for robots that are easy to sanitize for use in hospitals. It is also expected that this robot will be effective in reducing the fatigue of medical workers. In recent years, relatively inexpensive toys (e.g., Your Own Friend Doraemon with U, 21,780 yen including tax, https://www.takaratomy.co.jp/products/omnibot/doraemon_withu, etc.) have been sold and are popular among a wide range of generations. In this study, we decided to use a communication robot, which has the advantage of being easy to obtain and easy to take hygienic measures. The BOCCO emo LTE model (https://www.necolico.co.jp/emo/?utm_source=yahoo&utm_medium=cpc&utm_content=bocco&utm_campaign=202105_product&yclid=YSS.1001115894.EAIaIQobChMI77LontW89gIVWJNmAh18VAh3EAAYASAAEgI4C_D_BwE), a communication robot with a chat function released by Necolico, is available for rental as well as purchase. Since it is a robot, its mechanical appearance does not feel strange, and it is easy to disinfect. In addition, it has a cute character, and it is expected that patients will be interested and actively try to touch it. This robot was developed to allow family members who are far away to watch over the elderly, and has a chat function that can respond to greetings and notify schedules such as taking out the trash, as well as various environmental sensor functions. For patients with reduced activity, it is important to provide appropriate support, so we came up with the idea that this could be applied to delirium prevention measures.

The academic question of this study is whether communication robots can be an effective measure to prevent the onset of delirium. Previous research has shown the effectiveness of a team approach to delirium prevention measures for cancer patients1), and a new delirium high-risk patient care surcharge will be established from 2020. Research on the effect of the seal-shaped robot PARO on dementia care2), 3) has been conducted both domestically and internationally, and reports on different types of robots4) have also been published. The Ministry of Health, Labor and Welfare's website introduces the promotion of the development and dissemination of nursing robots (https://www.mhlw.go.jp/stf/seisakunitsuite/bunya/0000209634.html). On June 14, 2021, the Ministry of Health, Labor and Welfare launched a nursing robot needs and seeds matching support project (https://www.kaigo-ns-plat.com). The nursing robot portal site (http://robotcare.jp/jp/home/index.php) provides various information about robot nursing equipment, which is a subsidized project by the Ministry of Economy, Trade and Industry. The Japan Agency for Medical Research and Development has also taken up the Robot Nursing Equipment Development and Introduction Promotion Project as an all-Japan medical equipment development project (https://www.amed.go.jp/program/list/02/01/005.html). Applied research on nursing robots in clinical settings is an area where further development is expected.

The uniqueness of this research is that it is the first time that a communication robot is used to prevent the onset of delirium. Furthermore, as a specification suitable for clinical application, it uses a communication robot that is relatively inexpensive and easy to disinfect. The creativity of this research is that through this verification, the functions necessary to prevent the onset of delirium are clarified, and feedback from medical professionals is also utilized to develop a communication robot that is more suitable for clinical application in the future. We hope that this research will be the first step toward developing a communication robot suitable for actual clinical use, such as in acute care settings.

**2. Research objectives**

The objective of this study is to compare high-risk delirium patients who receive standard delirium countermeasures with those who receive the same countermeasures plus a communication robot to verify the effectiveness of the communication robot in preventing the onset of delirium. At the same time, if the subjects are dementia patients, we will investigate changes in behavioral and psychological symptoms. In addition, we will conduct a questionnaire survey of nurses who actually dealt with patients in this study to verify differences in the burden felt when using the robot and explore the functions required for a communication robot.

**3. Target patients and eligibility criteria**

The study will target high-risk delirium patients who meet the following inclusion and exclusion criteria.

(1) Inclusion criteria

① Patients who are judged to be at high risk for delirium in screening at the time of admission

In other words, patients who meet any of the following criteria

-70 years of age or older

-Organic brain disorder (including brain metastasis)

-Intensive care unit admission

-Physical restraint

-Dementia

-History of delirium

-Use of risky drugs (especially benzodiazepines)

-Having undergone or scheduled surgery requiring general anesthesia

② Patients who have been fully explained about participating in this study and have given their own voluntary written consent, or written consent from their family or representative

(2) Exclusion criteria

① Patients who have had psychiatric illness or symptoms prior to admission and whose symptoms are severe

② Heavy alcohol drinkers (1500ml of beer, 3 cups of sake, or 300ml of shochu per day for more than 5 years)

③ Patients who are judged by their attending physician to be difficult to participate in

④ Patients who are otherwise deemed unsuitable as subjects by the principal investigator

**4. Research Methods**

(1) Type and Design of Research

This is an intervention study conducted at Hitachi General Hospital alone. This is an open-label randomized controlled trial in which subjects are randomly assigned to a control group that receives regular delirium prevention measures and a group that receives the same measures plus a communication robot intervention, and the evaluation items are compared between the groups. In addition, after the subjects are discharged from the hospital, a questionnaire survey is conducted on the nurses who handled the patients in this study.

(2) Research Outline

Screening at admission identified patients as at high risk for delirium

Regular delirium prevention measures (control) group (72 patients)

Medical staff working in the ward implemented measures centered on non-pharmacological therapy using a team approach.

Regular measures + robot (intervention) group (72 people)

In addition to the same measures as the control group, a communication robot was installed and patients and their families were able to freely interact with it.

If the hospitalization period is less than 3 weeks,

the period until discharge.

If the hospitalization period is more than 3 weeks,

the period ends 3 weeks after hospitalization.

Evaluation

- Number of delirium cases using ICDSC

- If the patient has dementia, changes in behavioral and psychological symptoms using the Japanese version of DBD

- Feeling of burden on the nurse in charge through a questionnaire survey

**Control group:** The following measures are mainly non-pharmacological therapy using a team approach.

① Assistance with maintaining orientation for cognitive decline.

② Appropriate fluid replacement and water intake to treat and prevent dehydration.

③Gradual reduction and discontinuation of risky medications (especially benzodiazepines).

④ Efforts to encourage early mobilization.

⑤ Strengthening pain management, such as by using objective pain evaluations.

⑥ Appropriate sleep management, such as non-pharmacological promotion of sleep onset.

⑦ Provision of information about delirium to the patient and their family using pamphlets.

**Intervention group:** In addition to the same measures as in the control group, a Necorico communication robot, the "BOCCO emo LTE model", will be placed at the bedside. The principal investigator will perform the initial settings for the robot. The robot will be left at the bedside of the intervention group patients once settings have been completed, and will be available for the patient and their family to touch freely. During the intervention, nurses will disinfect the robot regularly every day. The principal investigator will also instruct ward nurses in each ward on how to use the robot, and will be able to contact them immediately by phone if a malfunction occurs with the robot.

(3) Rules for concomitant therapy

-No restrictions on any treatment administered by a physical physician.

(4) Case registration and allocation method

-Random allocation by ward using a random number table.

(5) Planned period of study participation

After consent, each subject will participate until discharge. However, if the hospitalization period is longer than three weeks, the study will end three weeks after admission.

**5. Observation and examination items**

Age, sex, diagnosis, department in charge, date of admission, date of discharge, and in the case of dementia, degree of independence in daily living, etc.

Delirium will be determined using the Japanese version of the Intensive Care Delirium Screening Checklist (ICDSC: Unoki, Mizutani, Sakuramoto).

For dementia patients, the Japanese version of the Dementia Behavior Disturbance Scale (DBD: Mizoguchi, Iijima) will be used to measure changes in behavior and psychological symptoms.

Observation and examination schedule

|  |  | Observation period (during hospitalization, but up to three weeks after hospitalization) | |  |
| --- | --- | --- | --- | --- |
| period | Before intervention | Date of admission | On the day of discharge or  3 weeks after admission | After discharge |
| Consent | ○ |  |  |  |
| Patient background | ○ |  | ○ |  |
| ICDSC |  | ←　○　→ | |  |
| DBD  (in case of dementia) |  | ○ | ○ |  |
| Adverse events |  | ←　○　→ | |  |
| Nurse questionnaire |  |  | | ○ |

**6. Expected benefits and disadvantages (side effects)**

(1) Expected benefits

There will be no direct benefit to the subjects from participating in this study. The results of the study may contribute to future medical advances.

(2) Expected disadvantages (side effects)

In both groups, it will take about 30 minutes for the explanation of the study and consent to be given. In the intervention group, although physical invasiveness is unlikely, it is possible that the robot's voice and movements may be uncomfortable. In addition, since the "BOCCO emo LTE model" is also an LTE wireless communication terminal, it cannot be used near medical equipment that may affect it. When using the robot, pay attention to the effects of malfunctions of medical equipment, etc., and observe the hospital's mobile phone handling speed, referring to the Ministry of Internal Affairs and Communications Radio Wave Use Website (https://www.tele.soumu.go.jp/j/sys/ele/index.html) and other sources.

**7. Evaluation Items (Endpoints)**

(1) Primary Evaluation Item

The number of cases of delirium during hospitalization up to 3 weeks after admission, as determined by the Japanese version of the ICDSC.

(2) Secondary Evaluation Item

1. In the case of dementia, changes in behavioral and psychological symptoms using the Japanese version of the DBD scale.

2. A questionnaire survey of nurses who actually handled patients in this study to determine the burden felt when using the communication robot and areas for improvement in the robot.

**8. Handling of Adverse Events**

For all adverse events (subjective symptoms and abnormal test results) that occurred from the start of intervention to the end of intervention, the symptoms, onset date, disappearance date, outcome, degree, treatment, and causal relationship to the communication robot will be determined and recorded in the case report.

**9. Discontinuation criteria for individual subjects**

(1) When consent is withdrawn by the patient, family member, or representative

(2) When a request to change or discontinue treatment is made by the patient, family member, or representative

(3) When it becomes impossible to place an LTE wireless communication terminal at the bedside

(4) When the attending physician judges it inappropriate to continue the study due to the occurrence of adverse events (worsening of the primary disease, worsening of complications, onset of a new disease, etc.)

(5) When the attending physician judges it inappropriate to continue the study for other reasons

(6) When the principal investigator judges it inappropriate to continue the study

**10. Study Period**

October 1, 2022 to March 31, 2025 (Registration Deadline: September 31, 2024)

**11. Target number of cases and statistical analysis method**

(1) Target number of cases

The target sample size is 72 cases in each group (total of 144 cases) when the chi-square test is performed with G*power, α=0.05, 1-β=0.8, and effect size is 0.3.

(2) Statistical analysis method

To calculate the incidence of delirium in each group, odds ratios are calculated and two-sided 95% confidence intervals are constructed. To examine intergroup comparisons, chi-square tests are performed with a two-sided significance level of 5%. Changes in behavioral and psychological symptoms of dementia before and after intervention are compared between groups using the Mann-Whitney test.

**12. Consideration of human rights of subjects and method of protecting personal information**

All personnel in this study will comply with the "Declaration of Helsinki (revised October 2008)" and the "Ethical Guidelines for Life Science and Medical Research Involving Human Subjects" announced in 2021.

When handling samples and other materials related to the research, they will be assigned numbers unrelated to the subjects' personal information and managed with due consideration given to protecting the subjects' confidentiality. When publishing the results of the research, information that can identify the subjects will not be included. Furthermore, the subjects' information obtained in the research will not be used for purposes other than the research.

**13. Response to and compensation for health damage to subjects**

If health damage occurs to a subject as a result of the implementation of this research, the researcher will take appropriate measures. In that case, if treatment or testing becomes necessary, it will be carried out within the subject's regular health insurance coverage. This point will be explained to the subject and their understanding will be obtained.

**14. Subjects' expenses**

The communication robot and its necessary expenses for this research will be covered by a Japan Society for the Promotion of Science research grant. All other matters will be covered within the regular health insurance coverage, so no expenses will be incurred by the subject as a result of participating in the research.

**15. Preservation of records and publication of research results**

The principal investigator will preserve important documents related to the implementation of the research (copies of application documents, notification letters from the hospital director, copies of various applications and reports, consent forms, and other documents or records necessary to ensure the reliability of the data, etc.) for five years after the research is discontinued or completed, and will then dispose of them with care to protect personal information.

The researcher will publish the results of this research by presenting them at relevant academic conferences, etc.

**16. Research funding and conflicts of interest**

This research will be conducted with funding from the Japan Society for the Promotion of Science. There is no conflict of interest between the researcher and the company (or related institution) that manufactures and sells the communication robot used in this research, as stated in the "Self-declaration of Conflicts of Interest in Clinical Research" in the separate application.

**17. Research implementation structure**

This research will be conducted under the following structure.

[Research participants]

○ Koubun Imai, Chief physician, Department of Mental Health, Hitachi General Hospital, Hitachi, Ltd.

Yumiko Matsumoto, Nurse, Ward 6, Nursing Department, Hitachi General Hospital, Hitachi, Ltd.

Naoyuki Kashimura, Social Worker, Social Welfare Counseling Office, Hitachi General Hospital, Hitachi, Ltd.

Sanae Shibata, Deputy Chief Nurse, Nursing Department, Hitachi General Hospital, Hitachi, Ltd.

(○ Research Director)

**18. Reference materials/literature list**

1) Ogawa A, Okumura Y, Fujisawa D, et al.: Quality of care in hospitalized cancer patients before and after implementation of a systematic prevention program for delirium: the DELTA exploratory trial. Support Care Cancer 2019; 27(2): 557-565

2) Takayanagi K, Kirita T, Shibata T: Comparison of verbal and emotional responses of elderly people with mild/moderate dementia and those with sever dementia in responses to seal robot, PARO. Front Aging Neurosci. 2014; 6: 257

3) Petersen S, Houston S, Qin H, et al.: The utilizattion of robotic pets in dementia care. J Alzheimers Dis. 2017; 55(2): 569-574

4) Valentí Soler M, Agüera-Ortiz L, Olazarán Rodríguez J, et al.: Social robots in advanced dementia. Front Aging Neurosci. 2015; 7: 133
