# Supplementary material for: Effect of a communication robot in the prevention of postoperative delirium in older persons: A randomized controlled trial
Source: PLoS One. 2025 Jul 29;20(7):e0327868. doi: 10.1371/journal.pone.0327868 (PMC12306737; doi:10.1371/journal.pone.0327868)
Supplement: S2 File — (DOC) [file pone.0327868.s005.doc]

自主臨床研究

**せん妄予防効果に関する**

**コミュニケーションロボットの介入研究**

**研　究　実　施　計　画　書**

研究責任者 日立総合病院　こころの診療科 今井　公文

作成日

2022年3月18日　計画書案　第0版作成

**目　次**

0．概要

1．研究の背景

2．研究の目的

3．対象患者および適格性の基準

4．研究の方法

5．観察および検査項目

6．予想される利益および不利益（副作用）

7．評価項目（エンドポイント）

8．有害事象発生時の取扱い

9．個々の被験者における中止基準

10．研究実施期間

11．目標症例数とその設定根拠および統計解析方法

12．被験者の人権に対する配慮および個人情報の保護の方法

13．被験者の健康被害への対応と補償

14．被験者の費用負担

15．記録の保存と研究結果の公表

16．研究資金および利益相反

17．研究実施体制

18．参考資料・文献リスト

**0．概要**

せん妄は、急激に発症して重症度が変動する注意および意識の障害であり、さらに認知の障害を伴う。落ち着かずに治療に拒否的となる場合もあり、入院期間が延長し、入院費用が増え、予後が不良となり、医療従事者も疲弊するなどの、様々な弊害が引き起こされて悪循環となりかねない。しかし、いまだにエビデンスのある治療法は無いため、有効な予防方法を探ることは喫緊の課題となっている。

本研究の目的は、せん妄ハイリスク患者に対して、通常のせん妄対策を行う群と、同じ対策にコミュニケーションロボットによる介入を加えた群とを比較し、コミュニケーションロボットのせん妄発症予防における有効性を検証することである。同時に、対象が認知症者である場合は行動・心理症状の変化を調査する。さらに、本研究で実際に患者対応を行った看護師にアンケート調査を行い、コミュニケーションロボットに求められる機能を探っていく。

対象は、入院時のスクリーニングでせん妄ハイリスクと判定されている患者のうち、入院前から重篤な精神症状を合併した患者やアルコール多飲者を除外したものとする。

研究は、日立総合病院単施設で行われる。通常のせん妄予防対策を行う対照群と、同じ対策におしゃべり機能付きのコミュニケーションロボット（ネコリコ社のBOCCO emo LTEモデル）による介入を加える群とに、無作為に割り付け、評価項目について群間比較を行う、非盲検のランダム化比較試験である。観察期間は、同意後から退院までであるが、入院期間が３週間以上となる場合は、入院後３週間の時点で終了とする。さらに、対象者の退院後、本研究で患者対応を行った担当看護師にアンケート調査を行う。

主要評価項目は、入院後最長3週間までの入院期間中のせん妄の発症件数であり、日本語版ICDSCを用いて判定する。副次的評価項目は、①認知症の場合は、日本語版DBD scaleを用いた、行動・心理症状の変化、②本研究で実際に患者対応を行った看護師へのアンケート調査による、コミュニケーションロボットを使用した時の負担感やロボットの改良点など、の２つである。

実施予定症例は、各群72例ずつ(合計144例)を目標数とする。研究期間は、2022年10月1日～2025年3月31日（登録締切日：2024年9月31日）の予定であるが、目標症例に達した時点で、登録は締め切ることとする。

**1．研究の背景**

せん妄は、急激に発症して重症度が変動する注意および意識の障害であり、さらに認知の障害を伴う。落ち着かずに治療に拒否的となる場合もあり、入院期間が延長し、入院費用が増え、予後が不良となり、医療従事者も疲弊するなどの、様々な弊害が引き起こされて悪循環に陥りかねないため、せん妄は急性期医療における重大な問題の一つである。研究責任者は総合病院の精神科医として長くせん妄対策に取り組んできたが、せん妄について実感するのは、せん妄にはいまだ有効な治療法は無く、発症予防がいかに重要であるかということであった。2020年度の診療報酬改定により「せん妄ハイリスク患者ケア加算」が新設され、せん妄ハイリスク患者のスクリーニング、非薬物療法を中心とした予防的対策、せん妄の早期発見などに対して、評価が始まった。しかし、いまだにエビデンスのある治療法は無いため、複数の予防的対策を組み合わせているのが現状であり、さらなる有効な予防方法を探ることは喫緊の課題となっている。

一方、かねてより経済産業省と厚生労働省において、介護ロボットの開発支援と実証が行われてきた。移乗介助、移動介助、排泄支援、入浴支援、認知症の方の見守りなどの分野で、介護ロボットの導入が検証されている。介護ロボットの中でもコミュニケーションを目的もしくは手段とするものが、コミュニケーションロボットである。高齢であることや認知症は、せん妄の代表的なリスク因子として知られている。アザラシ型ロボットPAROをはじめとしたぬいぐるみ型コミュニケーションロボットは、認知症ケアなどにおける有効性の研究が行われているところであり、臨床面への応用に対する期待も高い。しかし、PAROの価格は40万円程度と非常に高い。また、総じてぬいぐるみ型のロボットは、手触り感を重視するために消毒が困難であり、COVID-19問題も終息をみない現在、病院で使用するには衛生対策が容易なものが求められる。また、医療従事者の疲弊の軽減に対しても有効なロボットであることが期待される。

近年はおもちゃとして比較的安価なもの（例えば、キミだけのともだち ドラえもん with U、税込21,780円、<https://www.takaratomy.co.jp/products/omnibot/doraemon_withu>など）が販売され、幅広い世代に親しまれている。本研究でも、入手しやすく、かつ衛生対策が容易なメリットを持つ、コミュニケーションロボットを用いることとした。ネコリコ社から発売されたおしゃべり機能付きのコミュニケーションロボット「BOCCO emo LTEモデル」（https://www.necolico.co.jp/emo/?utm_source=yahoo&utm_medium=cpc&utm_content=bocco&utm_campaign=202105_product&yclid=YSS.1001115894.EAIaIQobChMI77LontW89gIVWJNmAh18VAh3EAAYASAAEgI4C_D_BwE）は、購入だけではなくレンタルでの利用も可能となっている。そもそもロボットの設定であるため、機械的な外観であっても違和感がなく、消毒は簡便である。また、可愛いキャラクターであり、患者側からも興味を持って積極的に触れようとすることが期待される。このロボットは、遠く離れた家族が高齢者を見守るために開発されており、例えば、あいさつに対して返事をしたりゴミ出しなどの予定を通知するなどのおしゃべり機能を持ち、各種の環境センサ機能も持っている。活動性の低下した患者に対しては、適度な働きかけを行うことも重要となるため、せん妄予防対策に応用できるのではないかと着想した。

せん妄の発症予防のために、コミュニケーションロボットが有効な対策となり得るか否かが、本研究の学術的「問い」である。これまでの研究では、癌患者に対してチームアプローチによるせん妄対策の有効性が認められ^1)^、2020年度からせん妄ハイリスク患者ケア加算が新設されることとなった。アザラシ型ロボットのPAROが認知症ケアに与える効果の研究^2), 3)^は国内外でなされており、ロボットのタイプ別の報告^4)^もなされている。厚生労働省のホームページでは、介護ロボットの開発・普及の促進について紹介されている(<https://www.mhlw.go.jp/stf/seisakunitsuite/bunya/0000209634.html>)。2021年6月14日からは、厚生労働省で介護ロボットのニーズ・シーズマッチング支援事業(https://www.kaigo-ns-plat.com)が開始となった。介護ロボットポータルサイト(<http://robotcare.jp/jp/home/index.php>)では、経済産業省の補助事業であるロボット介護機器に関する様々な情報が提供されている。日本医療研究開発機構でも、オールジャパンでの医療機器開発プロジェクトとして、ロボット介護機器開発・導入促進事業を取り上げている(<https://www.amed.go.jp/program/list/02/01/005.html>)。臨床場面における介護ロボットの応用研究は、さらなる発展が期待される領域である。

本研究の独自性は、まずせん妄の発症予防対策に、初めてコミュニケーションロボットを用いる点である。さらに臨床応用に適した仕様として、比較的安価で、消毒が簡便な、コミュニケーションロボットを用いる点にある。また、本研究の創造性は、この検証を通じてせん妄の発症予防のために必要な機能が明確化され、医療従事者からのフィードバックも生かし、より臨床応用に適したコミュニケーションロボットの開発へと将来的にも発展していくことである。本研究を、急性期医療現場などの実際の臨床に適したコミュニケーションロボットの開発につながる第一歩としたい。

**2．研究の目的**

本研究の目的は、せん妄ハイリスク患者に対して、通常のせん妄対策を行う群と、同じ対策にコミュニケーションロボットによる介入を加えた群とを比較し、コミュニケーションロボットのせん妄発症予防における有効性を検証することである。同時に、対象が認知症者である場合は行動・心理症状の変化を調査する。さらに、本研究で実際に患者対応を行った看護師にアンケート調査を行い、使用時の負担感の違いなどを検証し、コミュニケーションロボットに求められる機能を探っていく。

**3．対象患者および適格性の基準**

次の選択基準および選択除外基準を満たす、せん妄ハイリスク患者を対象とする。

（1）選択基準

①入院時のスクリーニングでせん妄ハイリスクと判定されている患者

　　すなわち、以下の項目のいずれかに該当した患者

　　　・70歳以上

　　　・脳器質的障害（脳転移を含む）

　　　・集中治療室入室

　　　・身体抑制

　　　・認知症

　　　・せん妄の既往

　　　・リスクとなる薬剤（特にベンゾジアゼピン系薬剤）の使用

　　　・全身麻酔を要する手術後又はその予定があること

②本研究への参加にあたり十分な説明を受けた後、十分な理解の上、患者本人の自由意思による文書同意、もしくは家族・代理権者からの文書同意が得られた患者

（2）除外基準

①入院前から精神病または精神症状を合併しており、かつ症状が重篤である

②アルコール多飲者である（1日ビール1500mlか日本酒3合か焼酎300mlを5年以上）

③担当医によって参加が困難と判断される

④その他、研究責任者が被験者として不適当と判断した患者

**4．研究の方法**

（1）研究の種類・デザイン

日立総合病院単施設で行われる介入研究である。通常のせん妄予防対策を行う対照群と、同じ対策にコミュニケーションロボットによる介入を加えた群とに、無作為に割り付け、評価項目について群間比較を行う、非盲検のランダム化比較試験である。さらに、対象者の退院後、本研究で患者対応を行った担当看護師にアンケート調査を行う。

（2）研究のアウトライン

入院時スクリーニングで、せん妄ハイリスク患者と判定

通常せん妄予防対策（対照）群（72名）

病棟に従事する医療スタッフが、チームアプローチによる非薬物療法を中心とした対策を行う。

通常対策＋ロボット（介入）群（72名）

対照群と同様の対策に加え、コミュニケーションロボットを設置し、患者や家族が自由に触れられるようにする。

　　　　　　　　　　　　　　　　　　　入院が3週間以内の場合は、退院までの期間

　　　　　　　　　入院期間が3週間以上の場合は、入院後3週の時点で終了

評価

・ICDSCを用いたせん妄の発症件数

・患者が認知症の場合は、日本語版DBDを用いた、行動・心理症状の変化

・アンケート調査による、担当看護師の負担感

**対照群**：チームアプローチによる非薬物療法を中心とした以下の対策を行う。

① 認知機能低下に対する、見当識の維持などの援助。

1. 脱水の治療・予防として、適切な補液と水分摂取。
2. リスクとなる薬剤（特にベンゾジアセピン系薬剤）の漸減・中止。
3. 早期離床の取り組み。
4. 痛みの客観的評価を併用するなど、疼痛管理の強化。
5. 非薬物的な入眠の促進など、適切な睡眠管理。
6. パンフレットを用いて、本人および家族へのせん妄に関する情報提供。

**介入群**：対照群と同様の対策に加え、ネコリコ社のコミュニケーションロボット「BOCCO 　emo LTEモデル」をベッドサイドに設置する。ロボットに対しては、研究責任者が初期設定を行う。設定が済んだ状態のロボットを、介入群患者のベッドサイドに介入終了時まで置き、患者や家族が自由に触れられるようにする。介入中は、看護師が毎日定期的にロボットの消毒を行う。また各病棟で病棟看護師に対して、研究責任者からロボットの使用法を教示し、ロボットに不具合が発生した際はすぐに電話にて連絡が入るようにする。

1. 併用療法についての規定

・身体科担当医が行ういかなる治療も制限しない。

（4）症例登録、割付の方法

・病棟ごとに乱数表による無作為割付。

（5）被験者の研究参加予定期間

各被験者は同意後、退院時まで参加する。ただし、入院期間が３週間以上となる場合は、入院後３週間の時点で終了とする。

**5．観察および検査項目**

年齢、性別、診断名、担当診療科、入院日、退院日、認知症の場合は日常生活自立度など。

日本語版ICDSC（Intensive Care Delirium Screening Checklist: 宇野木・水谷・櫻本）を用いて、せん妄を判定する。

認知症者には、日本語版認知症行動障害尺度（DBD [Dementia Behavior Disturbance] scale: 溝口・飯島）を用いて、行動・心理症状の変化を測定する。

観察および検査スケジュール表

|  |  | 観察期間 （入院中。ただし最長で入院後３週間） | |  |
| --- | --- | --- | --- | --- |
| 時期 | 介入前 | 入院日 | 退院日、または入院後３週時点 | 退院後 |
| 同意 | ○ |  |  |  |
| 患者背景 | ○ |  | ○ |  |
| ICDSC |  | ←　○　→ | |  |
| DBD（認知症の場合） |  | ○ | ○ |  |
| 有害事象 |  | ←　○　→ | |  |
| 看護師アンケート |  |  | | ○ |

**6．予想される利益および不利益（副作用）**

（1）予想される利益

本研究へ参加することによる被験者に直接の利益は生じない。研究成果により将来の医療の進歩に貢献できる可能性がある。

（2）予想される不利益（副作用）

いずれの群においても、研究に関する説明・同意の際に約30分の時間を要する。介入群においては、身体的な侵襲性は考えにくいものの、ロボットの話し声や動きを不快に感じる可能性はあると思われる。また、「BOCCO emo LTEモデル」はLTE方式の無線通信端末でもあるため、影響を与える医療機器のそばで使用することが出来ない。使用する際は、総務省電波利用ホームページ(<https://www.tele.soumu.go.jp/j/sys/ele/index.html>)などを参考に、医療機器の誤動作などの影響に注意を払い、病院内の携帯電話取り扱い気速を遵守する。

**7．評価項目（エンドポイント）**

（1）主要評価項目

日本語版ICDSCで判定する、入院後最長3週間までの入院期間中のせん妄の発症件数。

（2）副次的評価項目

①認知症の場合は、日本語版DBD scaleを用いた、行動・心理症状の変化。

②本研究で実際に患者対応を行った看護師へのアンケート調査による、コミュニケーションロボットを使用した時の負担感やロボットの改良点。

**8. 有害事象発生時の取り扱い**

介入開始から介入終了までに発生した全ての有害事象（自覚症状や検査値異常）について、症状・発症日・消失日・転帰・程度・処置・コミュニケーションロボットとの因果関係について判断し、症例報告書に記載する。

**9．個々の被験者における中止基準**

（1）患者あるいは家族・代理権者より同意の撤回があった場合

（2）患者あるいは家族・代理権者より治療の変更・中止の申し出があった場合

（3）LTE方式の無線通信端末をベッドサイドに置けなくなった場合

（4）有害事象の発現（原疾患の増悪、合併症の悪化、新たな疾患の併発等）により、担当医師が試験の継続を不適当と判断した場合

（5）その他、担当医師が試験の継続を不適当と判断した場合

（6）研究責任者が試験の継続を不適当と判断した場合

**10．研究実施期間**

2022年10月1日～2025年3月31日（登録締切日：2024年9月31日）

**11．目標症例数と統計解析方法**

（1）目標症例数

サンプルサイズは、G*powerにて、χ^2^検定、α=0.05、1-β=0.8、効果量0.3とした時の、各群72例ずつ(合計144例)を目標数とする。

（2）統計解析方法

各群のせん妄発生率を算出するためにオッズ比を算出しその両側95％信頼区間を構成する。群間比較を検討するため、χ^2^検定を両側有意水準5％で行う。介入前後での認知症の行動・心理症状の変化は、Mann-Whitney検定を行いて群間比較を行う。

**12．被験者の人権に対する配慮および個人情報の保護の方法**

本研究のすべての担当者は、「ヘルシンキ宣言（2008年10月修正）」および令和3年に告示された『人を対象とする生命科学・医学系研究に関する倫理指針』を遵守して実施する。

研究実施に係る試料等を取扱う際は、被験者の個人情報とは無関係の番号を付して管理し、被験者の秘密保護に十分配慮する。研究の結果を公表する際は、被験者を特定できる情報を含まないようにする。また、研究の目的以外に、研究で得られた被験者の情報等を使用しない。

**13．被験者の健康被害への対応と補償**

本研究の実施に伴い、被験者に健康被害が発生した場合は、研究担当者は適切な処置を講じる。その際、治療または検査等が必要となった場合は、被験者の通常の保険診療内で実施する。この点を被験者に説明し、理解を得ることとする。

**14．被験者の費用負担**

本研究のうち、コミュニケーションロボットとその必要経費は、日本学術振興会科学研究費で賄う。それ以外は通常の保険診療内で行われるため、研究に参加することによる被験者の費用負担は発生しない。

**15．記録の保存と研究結果の公表**

研究責任者は、研究等の実施に係わる重要な文書（申請書類の控え、病院長からの通知文書、各種申請書・報告書の控、同意書、その他データの信頼性を保証するのに必要な書類または記録等）を、研究の中止または終了後5年が経過した日までの間保存し、その後は個人情報に注意して廃棄する。

研究担当者は、本研究の成果を関連学会等において発表することにより公表する。

**16．研究資金および利益相反**

本研究は、日本学術振興会科学研究費で実施する。なお、この研究において用いるコミュニケーションロボットを製造販売している会社（または関連機関）と、研究者との間には、別途申請の「臨床研究の利益相反に関する自己申告書」のとおり、利害関係はない。

**17．研究実施体制**

本研究は以下の体制で実施する。

【研究分担者】

○ 今井　公文　　　株式会社日立製作所日立総合病院　こころの診療科　主任医長

松本　有美子　　株式会社日立製作所日立総合病院　看護局　本―６病棟看護師

樫村　直幸　　　株式会社日立製作所日立総合病院　社会福祉相談室　社会福祉士

柴田　早苗　　　株式会社日立製作所日立総合病院　看護局　副総看護師長

（○ 研究責任者）

**18．参考資料・文献リスト**

1) Ogawa A, Okumura Y, Fujisawa D, et al.: Quality of care in hospitalized cancer patients before and after implementation of a systematic prevention program for delirium: the DELTA exploratory trial. *Support Care Cancer* 2019; 27(2): 557-565

2) Takayanagi K, Kirita T, Shibata T: Comparison of verbal and emotional responses of elderly people with mild/moderate dementia and those with sever dementia in responses to seal robot, PARO. *Front Aging Neurosci*. 2014; 6: 257

3) Petersen S, Houston S, Qin H, et al.: The utilizattion of robotic pets in dementia care. *J Alzheimers Dis.* 2017; 55(2): 569-574

4) Valentí Soler M, Agüera-Ortiz L, Olazarán Rodríguez J, et al.: Social robots in advanced dementia. *Front Aging Neurosci*. 2015; 7: 133
