# Supplementary material for: Effect of a communication robot in the prevention of postoperative delirium in older persons: A randomized controlled trial
Source: PLoS One. 2025 Jul 29;20(7):e0327868. doi: 10.1371/journal.pone.0327868 (PMC12306737; doi:10.1371/journal.pone.0327868)
Supplement: Attachment — Submitted filename: Response_to_Reviewers.docx [file pone.0327868.s007.docx]

**Response to Reviewer #1**

I would like to express my appreciation to the Reviewers for their insightful comments, which have significantly helped me improve the paper. I have responded to each of your comments below.

**Comment 1:** However, it is not a case-control study as stated, but rather a randomized controlled trial. This needs to be addressed.

**Response:** I agree with your point. I have revised the title to reflect this. (P1 line 5)

**Comment 2:** I would be interested to understand why the authors chose to include only patients aged 70 and above, rather than starting at 65 years, which is more commonly used as the threshold for defining older adults.

**Response:** Thank you for raising this concern. I have explained the reason for using a 70-year-old benchmark in the revised manuscript. (P4 lines 83-88) Additionally, at Hitachi General Hospital, nurses routinely administer the Japanese version of the ICDSC to all newly admitted patients aged 70 years or older as part of their standard medical care.

**Response to Reviewer #2**

I would like to express my appreciation to the Reviewers for their insightful comments, which have significantly helped me improve the paper. I have responded to each of your comments below.

**Comment 1:** More clinical and/or demographic variables are needed in the Table 1 beyond the supplement 2 data check sheets. Future randomization certainly needs stratification as learned from the current information.

**Response:** I agree with your point. I have included some sentences in line with your point in the discussion and limitation paragraph. (P13 line 292-P14 line 297)

**Comment 2:** The limitations in the 'Discussion' should be expanded to alert the reader that the subset analysis p-values in Table 2 should be interpreted with caution as this involves multiple comparisons with no adjustment for the p-values.

**Response:** Thank you for your point. I have added a sentence alerting readers to interpret our results with caution, as pointed out. (P13 lines 285-286)

**Response to Reviewer #3**

I would like to express my appreciation to the Reviewer for their insightful comments, which have significantly helped me improve the paper. I have responded to each of your comments below.

**Title**

**Comment 1:** In the title, the term “efficacy” is used, whereas throughout the article, including in the study objective, the term “effect” is employed. Considering that these are similar but distinct concepts, it should be defined which term best aligns with the study’s aim. I suggest choosing “effect,” as it more closely reflects what is being proposed. Please standardize a single concept throughout the entire document.

**Response:** I agree with your comment. I have revised the title using effects,” as pointed out. (P1 lines 4-5)

**Comment 2:** I also recommend adding the target population of the study (elderly).

**Response:** I agree with your point. I have added the study population to the title, as suggested. (P1 lines 4-5)

**Introduction**

**Comment 3:** Concisely and objectively cite the specific outcome related to the focus “PARO” in individuals with dementia.

**Response:** Thanks for your suggestion. I have included the sentences pointing out the outcomes, as suggested. (P3 lines 64-66)

**Comment 4:** In the last paragraph, add that the study was conducted in patients in the postoperative period who received general anesthesia.

**Response:** Thanks for suggesting this. I have added this sentence to the manuscript in response to your point. (P4 lines 82-83)

**Materials and Methods**

**Comment 5:** The first paragraph has been removed (the objective should be maintained in the last paragraph of the introduction, as already present in the manuscript).

**Response:** I agree with your point. The first paragraph of Materials and Methods has been removed, and the objective was maintained in the last paragraph of the introduction. (P4 lines 93-94)

**Comment 6:** This study employs comparative groups (intervention and control) that have been randomized. Therefore, I understand that the study design is not case-control but a controlled randomized clinical trial. Please clarify this in the Materials and Methods section and update the title accordingly.

**Response:** Thanks for pointing this out. I have changed the title accordingly. (P1 lines 5)

**Comment 7:** The sample size calculation should be clearly explained. Was it based on a previous study? Did it consider an estimated dropout rate?

**Response:** There have been no previous studies using robots, nor have they been limited to advanced anesthesia surgery for patients 70 years of age or older. The incidence rate of delirium in all hospitalized patients is generally considered to be 10-30%, with the incidence rate increasing in elderly patients and those undergoing general anesthesia surgery. All patients in this study were hospitalized for scheduled surgery and did not include emergency admissions, allowing for the collection of a certain amount of medical record information in advance. Because the intervention of a communication robot is minimally invasive, there is little risk of dropout, and I predicted that 2 or 3 people would drop out.

**Comment 8:** It is not clear whether the objective of assessing safety was maintained after the change in the study design. To make the text more concise and fluid, I suggest maintaining this information only in the paragraph from line 96 to line 99, concluding after the word “trial.”

**Response:** Thank you for your suggestion. I have added a sentence about patient safety. (P5 lines 106-107)

**Participants**

**Comment 9:** Considering that in developed countries a person is generally regarded as elderly starting at 65 years old, why was the cutoff age set at 70 years or older?

**Response:** I have added the explanation to the revised manuscript. (P4 lines 83-88) Additionally, at Hitachi General Hospital, nurses routinely administer the Japanese version of the ICDSC to all newly admitted patients aged 70 years or older as part of their standard medical care.

**Comment 10:** Were only age and use of general anesthesia considered as inclusion criteria? Was a severity score, for example, taken into account? Could patients with neurological conditions, on mechanical ventilation, or with communication disorders be included?

**Response:** Although severity scores were not taken into consideration, I targeted patients who could understand the study's explanation and provide informed consent. In the case of patients with dementia, consent was obtained from both the patients and their families.

**Comment 11:** Please clarify whether all post-surgical destination units could be included and whether all types of surgery are eligible for inclusion.

**Response:** Thanks for your suggestion. I have added the text about inclusion criteria as pointed out. (P5 lines 115-117)

**Participant Recruitment**

**Comment 12:** Explicitly state that blinding was not employed and justify this choice, including how it constitutes a limitation of the study.

**Response:** I agree with your point. I have include the text about blinding as pointed out. (P6 lines 134-135). This is a pilot study, and in the future, it may be possible to conduct a seemingly identical blinded study using a different communication program.

**Comment 13:** In the last paragraph, lines 126/127, clarify whether patients could have been included without considering a minimum length of hospital stay.

**Response:** Thanks. I have added the sentence as suggested. (P6 lines 135-136)

**Data Collection**

**Comment 14:** If feasible, collect data on years of schooling, preoperative functional status prior to the hospitalization that motivated the admission, and the type and duration of delirium manifestation. If this is not possible, include it as a limitation of the study.

**Response:** Thanks for your suggestions. Duration of delirium was recorded in Supporting Information S2. I have added the sentence as pointed out. (P13 line 292-P14 line 294).

**Routine Standard of Care**

**Comment 15:** Improve the description of approaches for cognitive decline. Specify the content of this intervention, including activities or strategies used to maintain or stimulate cognitive functions.

**Response:** I have added the text on how we stimulated cognitive functions, as pointed out. (P7 lines 150-151)

**Comment 16:** Describe the professionals involved in the early mobilization protocol (e.g., physiotherapist, occupational therapist, speech therapist, etc.), including the frequency and intensity of mobilization activities.

**Response:** I have added the explanation of the professionals involved in this study, as suggested. (P7 lines 153-155)

**Comment 17:** Besides the pain assessment tool, specify the interventions used for pain management, including the name of the assessment tool cited in the text and additional pain management strategies (e.g., analgesic protocols, positioning, etc.).

**Response:** Pain management methods varied depending on the department (e.g., orthopedics, surgery, etc.) and were implemented differently in each inpatient ward.

**Comment 18:** What non-pharmacological methods are employed to promote sleep hygiene and management? Specify the techniques or strategies used (e.g., environmental modification, sleep protocols, relaxation techniques).

**Response:** Thank you for the questions and for pointing this out. I have added the texts specifying the techniques in the revised manuscript. (P7 line 157)

**Comment 19:** Who is responsible for delivering educational measures about delirium, and what is the content of this information? Specify the method used (e.g., verbal education, printed material, audiovisual aids).

**Response:** Thank you for the questions. I have revised the manuscript to show that the patient’s nurses delivered education orally and through printed materials, as you have pointed out. (P7 lines 158-159)

**Comment 20:** Indicate whether tools such as clocks and/or calendars are available in the bed/room to assist with orientation.

**Response:** Thanks. I have added the texts of tools that could assist with orientation, available in the room, to the revised manuscript. (P7 lines 150-151)

**Communication Robot Intervention**

**Comment 21:** Suggest including a photograph of the “BOCCO emo LTE” within the context of hospitalization, in accordance with the journal’s policies regarding images and photographs.

**Response:** I have added the photograph as appendix S3.

**Comment 22:** Specify that interaction could be carried out verbally and through physical contact.

**Response:** I have added that patients and their family members could interact with the robot through physical contact. (P7 lines 163-164)

**Comment 23:** Clarify whether the researcher and/or healthcare professional instructed patients and/or family members to interact with the robot, and if so, how frequently this occurred.

**Response:** No, although the functions of the robot were explained to the patients and their caregivers, they were not instructed to interact with the robot, leaving the decision to their discretion. I have included this explanation in the revised manuscript. (P7 lines 168-170)

**Comment 24:** Explain whether there were any interaction barriers when the patient approached the robot, or if all responses were possible regardless of the patient's approach.

**Response:** The interaction barriers have been added to the revised manuscript. (P7 lines 170-172)

**Comment 25:** In cases of urgency or emergency, could the robot, if verbally activated by the family or patient, play any role in activating codes or alerting the nursing team?

**Response:** Not at all; the robots do not alert the nursing team. I have added this barrier to the revised manuscript. (P7 lines 172-174)

**Comment 26:** Consider providing an example of how the robot could interact based on a patient-initiated interaction.

**Response:** I have added the forms of patient-robot interactions to the manuscript, as suggested. (P7 lines 165-167)

**Comment 27:** Indicate whether the robot is stationary, fixed in a single location, or if it has mobility. Make this clear.

**Response:** The robot could move. I have added an explanation about this to the revised manuscript. (P7 lines 164-165)

**Comment 28:** Was the level of patient-robot interaction measured? If not, note this as a limitation of the study.

**Response:** The level of patient-robot interaction was not measured. I have included this in the limitation. (P14 lines 297-299)

**Delirium Assessment**

**Comment 29:** Why was the "Japanese version of the Intensive Care Delirium Screening Checklist" selected? The name suggests its applicability in ICU settings. Was it used regardless of the patient’s admission unit in the postoperative period?

**Response:** I have included the reason for this in the revised manuscript, as pointed out. (P8 lines 195-199) I adopted the Japanese version of ICDSC because the study was carried out in Japan.

**Comment 30:** Include information on the reliability, validity, sensitivity, and specificity of this assessment tool for delirium diagnosis.

**Response:** I have added this information to the revised manuscript (P8 lines 194-195), as identified in the published work in reference [14].

**Comment 31:** Although nursing staff recorded the occurrence of delirium in a spreadsheet between 6:00 a.m. and 6:00 a.m., it is unclear how frequently the scale was applied during this period (i.e., how many times per day delirium was screened).

**Response:** I have added the sentence with the answer to this question in the manuscript. (P9 lines 201-202)

**Statistical Analysis**

**Comment 32:** Both in the statistical analysis (line 175) and in the study objective (line 91), the author proposes to assess the effect of the robot on the incidence of delirium; however, the results are presented as prevalence (line 185).

It is understood that if the study aims to identify the occurrence of delirium over the entire hospitalization period, the incidence should be measured. Therefore, the way the results are presented needs to be adjusted accordingly. Although the “Delirium Assessment” section clearly states that the proposal is to identify the occurrence of delirium throughout hospitalization, in the Methods section (lines 87-88), the objective contradicts this, leading the reader to believe that the goal is to reduce the onset (which would relate to prevalence).

Please make these adjustments throughout the document to ensure consistency and align the results presentation with the stated objective. This lack of standardization was also observed in the discussion (lines 245-246) and the conclusion (line 255).

**Response:** Thank you so much for your comment. I have replaced "prevalence" and "onset" with "incidence throughout the manuscript."

**Comment 33:** It is necessary to clarify whether the intention-to-treat approach was used. If it was not, this should be explicitly mentioned as a limitation of the study.

**Response:** Thanks for your suggestion. I have added the text to the manuscript, as pointed out. (P9 lines 202-203)

**Comment 34:** I suggest including an Odds Ratio analysis to evaluate the association between variables.

**Response:** Logistic regression analysis was not performed in this study. The calculable odds ratios are included in Table 2.

**Participant Characteristics**

**Comment 35:** Begin the presentation of the results by describing the number of patients screened (which is not included in the flowchart and should also be added), those eligible for study inclusion, and those excluded.

**Response:** Thanks for pointing this out. I have revised Figure 1 to reflect this change. (P9 lines 214-217)

**Comment 36:** In the characteristics section, it is necessary to clearly state whether there were significant differences in clinical and demographic characteristics between the groups.

**Response:** Thanks for the suggestion. I have added the text showing the significant differences to the result section and Table 1. (P9 lines 221-223)

**Comment 37:** It is not necessary to include the IQR and the absolute and relative frequencies (% estimates) in the text; these details should be left exclusively in the table.

**Response:** I have revised the sentences to reflect your suggestions. (P9 lines 218-221)

**Prevalence of Delirium and Associated Risk Factors**

**Comment 38:** As previously mentioned, if the objective is to assess the incidence, the results in this section should be adjusted accordingly.

**Response:** I have adjusted the results to address your comments 39 to 43 as much as possible.

**Comment 39:** Once the incidence is measured, include the time taken for delirium onset in each group.

**Response:** The time from surgery to the incidence of delirium was recorded in days, not hours. Most cases occurred on the day of surgery, and the time differences for each factor were unclear. The number of days from surgery to the onset of delirium was described. (P10 lines 238-242)

**Comment 40:** It is not necessary to include the IQR or the absolute and relative frequencies (%). These details should be exclusively presented in the table.

**Response:** I have changed the sentences to reflect your point. (P9 lines 218-221)

**Comment 41:** There are two repeated paragraphs between lines 186-188 and lines 200-202; they should be consolidated or removed.

**Response:** You are right. The distinction between the main text and the table attachments was unclear, so I have moved them. (P9 lines 231-234)

**Comment 42:** Add a column with Odds Ratios in Table 2 to evaluate the association between the variables.

**Response:** Logistic regression analysis was not performed in this study. The calculated odds ratios are listed in Table 2.

**Comment 43:** Include in the results which type of surgery was most frequent and whether the surgeries were elective or emergency.

**Response:** I have added the most common type of surgery, as pointed out. (P10 lines 223-224). Moreover, in the "Participants subsection," I have added the sentence that emergency surgeries were not included. (P5 lines 111-112)

**Discussion**

**Comment 44:** Write a brief paragraph justifying how there were no patient losses throughout the trial.

**Response:** I have added the reason that I did not include emergency surgery to the revised manuscript. (P13 lines 278-279)

**Comment 45:** Lines 222 and 245: define whether the focus is on effect or effectiveness, as previously mentioned.

**Response:** Thank you for your comment. The focus of the study is effect, not effectiveness.

**Comment 46:** Line 230: clarify whether the patient’s delirium history refers to the current hospitalization or another context/moment.

**Response:** Thank you for your comment. A patient's delirium history refers to their previous history, not their current hospital stay. (P13 line 272)

**Comment 47:** A reflection could be included on whether humanization in healthcare and patient-centered care goals with therapies are feasible or not when using robotic systems.

**Response:** I have added this reflection to the revised manuscript. (P13 lines 268-270)

**Comment 48:** Add that this study had limited control over confounding variables and low rigor in the inclusion criteria for patients.

**Response:** I have added these points to the limitation paragraph in the revised manuscript, as pointed out. (P14 lines 294-296)
